# Multiple Coronary Artery Thrombosis in 5,10-Methylenetetrahydrofolate Reductase Gene Mutation

**DOI:** 10.4061/2011/856479

**Published:** 2011-09-13

**Authors:** Alfonso Campanile, Fabiola B. Sozzi, Gian Battista Danzi

**Affiliations:** Department of Cardiology, Fondazione IRCCS Cà Granda, Ospedale Maggiore Policlinico, Via F. Sforza, 35 20122 Milano, Italy

## Abstract

A 42-year-old man presented at our attention with chest pain. His cardiac risk factors were smoking habit and family history of coronary artery disease. At the ECG, a mild ST-segment elevation in the inferior leads was shown. A normal left ventricular function was demonstrated at the echocardiography. An emergency coronary angiography was performed, and an extensive thrombosis of the right coronary artery and midleft anterior descending coronary artery was visualized. A primary angioplasty with thrombus aspiration and direct stenting of both sites followed. Biochemical analysis revealed a high plasma homocysteine level with a homozygotic anomaly of the 5,10-methylenetetrahydrofolate reductase. Currently, a nine-month followup negative for cardiac events is recorded.

## 1. Introduction

Simultaneous thrombosis of multiple epicardial coronary arteries is an uncommon angiographic finding in ST-segment elevation myocardial infarction. It is related to several factors such as diffuse vessel spasm, state of hypercoagulability, and a decreased coronary blood pressure [1].

Hyperhomocysteinemia is a risk factor of coronary artery disease associated to arterial thrombosis. It is characterized by an abnormally high concentration of homocysteine (a sulfurated amino acid produced through the methionine metabolism). 

This paper describes a case of ST-segment elevation myocardial infarction secondary to thrombosis of two coronary arteries in a patient affected by intermediate hyperhomocysteinemia gene mutation related.

## 2. Case Presentation

**Figure 1:**
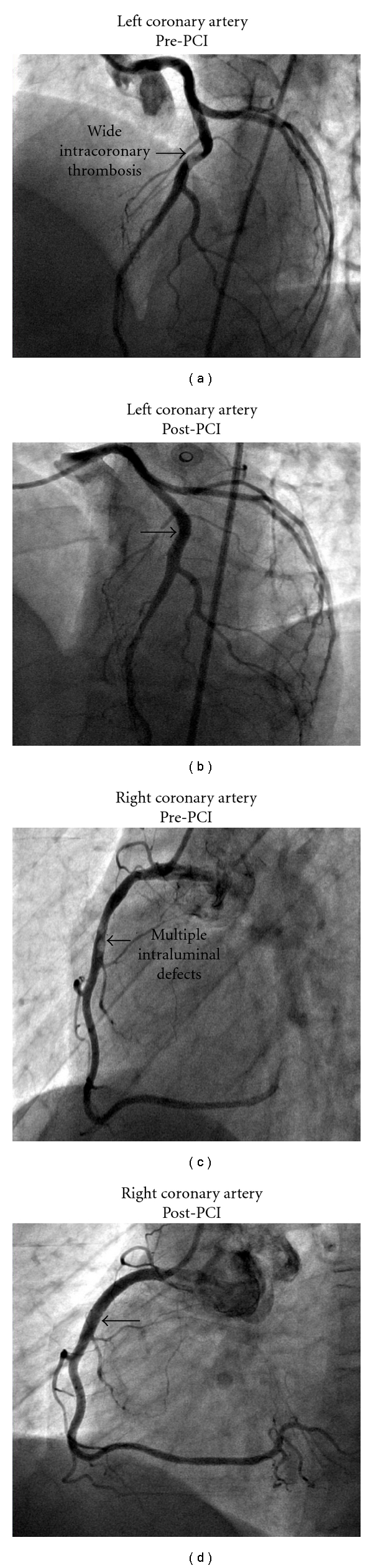


A 42-year-old male presented at the emergency room of the Hospital Policlinico, Milan, Italy with chest pain. He did not have any medical disease including hypertension, diabetes mellitus, dyslipidemia, and denied cocaine abuse. He was a smoker with familial premature coronary artery disease history; his body mass index was 22.6. The vital signs were normal (blood pressure: 130/80 mmHg, heart rate: 73 bpm, and oxygen saturation: 100%), and physical examination revealed no abnormalities. A 2 mm ST-segment elevation in the inferior leads was documented at the electrocardiogram. After an initial medical treatment with unfractionated heparin, acetyl salicylic acid, and clopidogrel, the patient was transported to the catheterization laboratory. The coronary angiogram revealed a normal left main and circumflex artery and two luminal irregularities of right coronary artery (RCA) and left anterior descending artery (LAD) complicated by wide coronary thrombosis (Figures 1(a) and 1(c)). Then, a glycoprotein IIb/IIIa inhibitor was administered according to standard protocol and thrombus aspiration, followed by direct stent implantation in the mid-RCA and mid-LAD (resp., with two and one bare metal stent), which were successfully performed (Figures 1(b) and 1(d)). A transthoracic echocardiogram showed a mildly reduced left ventricular function (ejection fraction 53%) with a normal right ventricular size and function.

A moderately high plasma homocysteine level (>50 *μ*mol/L) with normal serum vitamin B12 and folate levels were found at the biochemical analysis, together with normal renal and serum lipid profiles. A mild increase of troponin T (peak 0.31 ng/mL, normal range: 0.00–0.03) was detected. The thrombophilia screening findings are shown in Table 1. The genetic testing for the methylenetetrahydrofolate reductase showed the presence of C677T mutation in homozygosis. Therefore, oral folic acid supplementation (5 mg/die) was initiated. Currently, a nine-month followup negative for cardiac events is recorded; the plasma homocysteine level remained at the upper normal limit after methionine treatment.

## 3. Discussion and Conclusion

Simultaneous multivessel thrombosis in the setting of acute myocardial infarction is a rare entity. A case of acute thrombosis of two simultaneous coronary arteries in a young adult with familial hyperhomocysteinemia is described. 

Numerous studies have indicated that an increased plasma homocysteine level is a risk factor for occlusive arterial or venous disease [2–4]. 

Hyperhomocysteinemia usually occurs in people with at least one defective gene that affects the breakdown of homocysteine. There are two common defective genes in the population. The first one is related to the enzyme methylenetetrahydrofolate reductase, the second to the methioninesynthetase. Other causes of hyperhomocysteinemia are represented by deficiency of vitamin B, impaired renal function, and negative lifestyle factors (smoking habit, coffee abuse, and sedentary lifestyle). [5, 6] Hyperhomocysteinemia is classified as mild-moderate (15–30 *μ*mol per liter), intermediate (>31–100 *μ*mol per liter), and severe (>100 *μ*mol per liter) [7]. 

Subjects with moderate hyperhomocysteinaemia are characterized by a prothrombotic, and dysfibrinolytic state and homocysteine level is an independent predictor of thrombotic events. [8] The oxidative injury of endothelium in homocysteinaemia, combined with the lack of vasculoprotective effects of nitric oxide, predisposes to thrombotic events [8]. It is now widely accepted that increased plasma homocysteine is associated with increased cardiovascular risk independently of other atherosclerosis risk factors. [9] However, it is still unclear whether plasma homocysteine can be approached as a modifiable risk factor for atherosclerosis [10]. Our patient showed elevated homocysteine levels associated with homozygosity for the C677T gene mutation of methylenetetrahydrofolate reductase. A significantly higher frequency of this genetic condition was found in patients with early coronary artery disease onset (age <45 years) [11]. 

Politi et al. presented a case of a 35-year-old patient, with extensive anterior AMI and multiple thrombotic occlusion (in the distal LAD, diagonal branches, and obtuse marginal branch). Also this case was associated to elevated homocysteine levels (>50 *μ*mol/L) with normal thrombophilic and lipide profile [12].

No routine screening for elevated homocysteine and treatment is currently recommended except in patients who have premature atherosclerosis [13]. 

Further investigation on hyperhomocysteinaemia and coronary heart disease is needed.

## Figures and Tables

**Table 1:** Summary of thrombophilia screening laboratory findings.

Tests	Results (normal range)
Prothrombin time	10.80 seconds (8.9–11.7)
International normalized ratio (INR)	0.96 (0.90–1.14)
Activated partial thromboplastin time	25.7 (24.5–35.2)
Homocysteine (Hcy)	>50 *μ*mol/L (4.00–15.40)
Protein C activity (%)	122% (72–160)
Protein S activity (%)	152% (79–183)
Antithrombin III activity	108.0% (82.0–112.0)
Factor V Leiden mutation	Negative
Prothrombin gene mutation	Negative
Phospholipid (cardiolipin) AB IgG	1.8 U/mL GPL (0–10)
Phospholipid (cardiolipin) AB IgM	0.0 U/mL MPL (0–10)
Lupus anticoagulant	Absent
